# Adipocyte-Derived Extracellular Vesicles Endow Melanoma Cells with Stem-like Traits via PGC-1α–Mediated Mitochondrial Reprogramming

**DOI:** 10.3390/antiox15030333

**Published:** 2026-03-06

**Authors:** Gaia Giannitti, Sara Marchesi, Riccardo Garavaglia, Ivan Preosto, Emanuela Carollo, Patrizia Sartori, Fabrizio Fontana

**Affiliations:** 1 Department of Pharmacological and Biomolecular Sciences “Rodolfo Paoletti”, University of Milan, 20133 Milan, Italy; gaia.giannitti@unimi.it (G.G.); sara.marchesi31@gmail.com (S.M.); riccardo.garavaglia2@studenti.unimi.it (R.G.); ivan.preosto@studenti.unimi.it (I.P.); 2 Department of Biological and Medical Sciences, Oxford Brookes University, Oxford OX3 0BP, UK; emanuela.carollo2@gmail.com; 3 Department of Biomedical Sciences for Health, University of Milan, 20133 Milan, Italy; patrizia.sartori@unimi.it

**Keywords:** melanoma, adipocytes, extracellular vesicles, cancer stem cells, mitochondria, ROS

## Abstract

Melanoma is an aggressive cancer characterized by a rapid metastatic process. Thus, understanding the mechanisms underlying its progression is urgently needed to improve patient outcomes. In this regard, there is consistent evidence of a tumor-sustaining crosstalk between melanoma and subcutaneous adipose tissue; however, the role of extracellular vesicles (EVs) in this communication still needs to be clarified. We demonstrated that the EVs derived from adipocytes did not alter melanoma cell proliferation but significantly promoted tumor cell migration and invasion by determining an enrichment in mesenchymal markers, such as N-cadherin and vimentin. In particular, these changes were accompanied by the transition towards a stem-like phenotype, characterized by enhanced spherogenic ability and ABCG2 upregulation; interestingly, this led to a reduced in vitro response to the BRAF inhibitor vemurafenib. Mechanistically, an increase in PGC-1α expression was found, resulting in higher mitochondrial mass and activity, ATP synthesis, and ROS overproduction; of note, treatment of melanoma cells with SR-18292 and XCT790, two inactivators of mitochondrial biogenesis, and N-acetylcysteine, a ROS scavenger, successfully counteracted the above EV-related effects, suggesting that mitochondrial function could be targeted to suppress the vesicular interactions between adipose tissue and melanoma. Taken together, these results highlight the crucial role played by EVs in melanoma stroma, pointing out the ability of adipocyte-derived vesicles to sustain cancer aggressiveness via PGC-1α–dependent mitochondrial reprogramming.

## 1. Introduction

Cutaneous melanoma represents the most lethal form of skin cancer, accounting for the vast majority of skin cancer-related deaths despite representing only a fraction of all skin cancer diagnoses [1,2]. It arises from the malignant transformation of melanocytes, the pigment-producing cells of the epidermis, and is distinguished by its remarkable capacity for aggressive growth and dissemination [3,4]. The natural evolution of melanoma typically unfolds in distinct clinical and histopathological stages, ranging from localized and noninvasive lesions (stages 0 and I), in which malignant cells are confined to the epidermis or superficial dermis, to advanced disease (stages III and IV), characterized by regional lymph node involvement and distant metastases [5,6]. A key turning point in melanoma progression is the transition from a radial to a vertical growth phase, during which tumor cells penetrate deeper into the dermis [6]. Through this vertical expansion, melanoma cells gain access to subcutaneous tissue, a compartment largely populated by adipocytes [7,8]. Once within this environment, malignant cells acquire the capacity to intravasate into the lymphatic or vascular systems, thereby seeding systemic metastases [7,9]. Given this trajectory, patient survival is tightly linked to early detection, highlighting the critical importance of prompt and accurate diagnosis [10,11]. Nevertheless, even with advances in diagnostic imaging and molecular profiling, therapeutic options for advanced melanoma remain limited, and novel pharmacological targets capable of halting tumor progression are urgently needed [5,6,12,13].

In recent years, extracellular vesicles (EVs) have emerged as a central focus in cancer biology [14,15,16]. EVs constitute a heterogeneous group of membrane-bound, nanoscale particles, including exosomes, microvesicles, and apoptotic bodies, that are released by virtually all cell types [17,18]. These vesicles are widely distributed in biological fluids such as blood, lymph, and interstitial fluid, where they function as potent mediators of intercellular communication [19]. By transporting bioactive cargoes composed of DNA fragments, coding and non-coding RNAs, proteins, and lipids, EVs influence the behavior of recipient cells and orchestrate both physiological and pathological processes [17,18,19]. Importantly, EVs are now recognized as abundant and functionally active components of the tumor microenvironment, where they not only sustain tumor growth but also contribute to immune evasion, angiogenesis, and metastasis [20,21].

Among the stromal elements of the tumor microenvironment, adipocytes of the hypodermis have recently garnered attention for their dynamic role in supporting melanoma progression [8]. Far from being passive energy storage cells, adipocytes actively secrete metabolites, adipokines, and inflammatory cytokines that can reprogram cancer cell metabolism and enhance their invasive potential [22,23,24,25,26]. These observations have fueled growing interest in the crosstalk between adipocytes and melanoma cells [27]. However, despite the accumulating evidence on soluble mediators, the contribution of EVs derived from subcutaneous adipose tissue to melanoma progression remains poorly understood [8,27]. Whether adipocyte-derived EVs act as critical vectors of pro-tumorigenic signals or represent potential therapeutic vulnerabilities has not yet been fully elucidated.

In the present study, we sought to clarify the molecular mechanisms underpinning the interaction between adipocytes and melanoma cells, with a particular focus on the role of EVs in modulating malignant traits. By investigating the EV-mediated exchange of bioactive molecules within the adipose–melanoma axis, we aim to provide novel insights into how these vesicles shape the tumor microenvironment and drive disease progression. Our findings may not only deepen the understanding of melanoma biology but also uncover promising avenues for the development of innovative therapeutic strategies targeting EV-mediated communication.

## 2. Materials and Methods

### 2.1. Chemicals

For flow cytometry, the ABCG2 antibody (NB110-93511AF405) was purchased from Biotechne, Minneapolis, MN, USA.

The primary antibodies Alix (2171), CD9 (98327), N-cadherin (13116), vimentin (5741), and GAPDH (5174) were from Cell Signaling Technology Inc. (Danvers, MA, USA). TSG101 antibody (ab30871) was from Abcam (Cambridge, UK). Hsc70 (13D3) and calnexin (AF18) antibodies were from Thermo Fisher Scientific (Waltham, MA, USA). Cytochrome c antibody (sc-13560) was from Santa Cruz Biotechnology Inc. (Santa Cruz, CA, USA). PGC-1α (ab110411) was from Abcam, (Cambridge, UK). All the antibodies were diluted 1:1000 for experimental use.

Horseradish peroxidase (HRP)-conjugated secondary antibodies were from Cell Signaling Technology Inc., and enhanced chemiluminescence (ECL) reagents were from Cyanagen (Bologna, Italy).

SR-18292, XCT790, and N-acetylcysteine (NAC) were from Sigma-Aldrich, Milan, Italy.

### 2.2. Cell Lines and Cell Culture

A375 and WM115 melanoma lines, along with 3T3-L1 pre-adipocytes, were obtained from the ATCC (Manassas, VA, USA). Cells were maintained in DMEM supplemented with 10% FBS, glutamine, and antibiotics at 37 °C in a 5% CO_2_ environment. Cells were thawed from liquid nitrogen and maintained in the appropriate medium for 10–12 weeks. Subculturing was performed weekly using trypsin-EDTA to detach cells.

### 2.3. 3T3-L1 Cell Differentiation

As previously reported [28,29,30], 3T3-L1 cells were treated with a cocktail consisting of 500 μM IBMX, 1 μM dexamethasone, 1 μg/mL insulin, and 1 μM rosiglitazone in 10% FBS/DMEM to induce differentiation. After 72 h, the medium was transitioned to DMEM with 10% FBS and 1 μg/mL insulin for 4 days, followed by 3 days in standard growth medium prior to further experiments.

### 2.4. Extracellular Vesicle Isolation

FBS was subjected to ultracentrifugation at 120,000× *g* for 16 h to remove endogenous EVs. The resulting EV-free serum was then supplemented into DMEM to obtain EV-depleted medium (EDM). Cells were maintained in EDM for 48 h prior to EV collection. Conditioned media were processed for EV isolation using size-exclusion chromatography (SEC). Briefly, media were sequentially centrifuged at 300× *g* for 5 min and 16,500× *g* for 20 min at 4 °C to eliminate cells and debris. The clarified supernatant was concentrated by ultrafiltration through a 100 kDa polyethersulfone membrane (Vivaspin, Sigma-Aldrich) at 3000× *g*. A volume of 0.5 mL was applied to a Sepharose-based SEC column (Bio-Rad Laboratories Inc., Hercules, CA, USA), eluted with PBS, and fractions 7 to 11 were collected and combined. The pooled fractions were further concentrated using a 5 kDa PES filter to a final volume of 100 μL. Purified EVs were stored at −80 °C until use.

### 2.5. Nanoparticle Tracking Analysis

EV size distribution and concentration were evaluated using a NanoSight LM10 instrument with nanoparticle tracking analysis (NTA) 2.0 software (Malvern Instruments, Malvern, UK). For each sample, five 30 s videos were captured, and mean particle diameter (nm) and concentration (×10^8^ particles/mL) were calculated. All analyses were performed in duplicate.

### 2.6. Transmission Electron Microscopy

6 μL of EV suspension (30 μg/mL) was applied to Formvar carbon-coated 300-mesh copper grids. After 3 min, excess liquid was removed, and grids were stained with 2% aqueous uranyl acetate for 1 min. Grids were briefly rinsed in distilled water, blotted, air-dried, and imaged using a Zeiss EM10 transmission electron microscope (Carl Zeiss, Oberkochen, Germany).

### 2.7. Cell Proliferation Assay

Melanoma cells were seeded in 6-well plates at 5 × 10^4^ cells per well. After 24 h, they were incubated with adipocyte-derived EVs (30 μg/mL) for 96 h. Cells were then collected and counted using a Luna automated cell counter (Logos Biosystems, Annandale, VA, USA) following staining with 0.4% Trypan Blue (1:1 *v*/*v*), as previously reported [31,32].

### 2.8. Transmigration Assay

Melanoma cells were seeded in 6-well plates at 5 × 10^4^ cells per well. After 24 h, they were incubated with adipocyte-derived EVs (30 μg/mL) for 24 h. Cells were then collected and placed at 1 × 10^5^ cells per well into the upper chamber of 24-well transwell plates with 8 μm-pore membranes; complete medium was placed in the lower chamber. After 6 h, migrated cells on the underside of the membrane were fixed, stained with Diff-Quick solution (DADE, Düdingen, Switzerland), and quantified by counting three randomly selected microscopic fields.

### 2.9. Invasion Assay

Melanoma cells were seeded in 6-well plates at 5 × 10^4^ cells per well. After 24 h, they were incubated with adipocyte-derived EVs (30 μg/mL) for 24 h. Cells were then collected and placed at 1 × 10^5^ cells per well into the upper chamber of 24-well transwell plates with Matrigel-coated 8 μm-pore membranes; complete medium was placed in the lower chamber. After 12 h, migrated cells on the underside of the membrane were fixed, stained with Diff-Quick solution, and quantified by counting three randomly selected microscopic fields.

### 2.10. Sphere Formation Assay

Melanoma cells were seeded in 6-well plates at 5 × 10^4^ cells per well. After 24 h, they were incubated with adipocyte-derived EVs (30 μg/mL) for 24 h. Cells were then collected, placed at 2 × 10^5^ cells in 25-cm^2^ flasks, and cultured under non-adherent conditions in Euromed-N medium (Euroclone, Pero, Italy) supplemented with 10 ng/mL EGF, 10 ng/mL FGF2, and 1% N2 supplement (Thermo Fisher Scientific) for 7 days to determine their spherogenic ability. Formed spheroids were imaged and counted using a Zeiss Axiovert 200 microscope (Zeiss, Oberkochen, Germany) with a 10×/1.4 objective and Coolsnap Es CCD camera.

### 2.11. Determination of ABCG2 Enrichment

Melanoma cells were seeded in 6-well plates at 5 × 10^4^ cells per well. After 24 h, they were incubated with adipocyte-derived EVs (30 μg/mL) for 24 h. Cells were then collected, placed at 2 × 10^5^ cells in 25-cm^2^ flasks, and cultured under non-adherent conditions in Euromed-N medium supplemented with 10 ng/mL EGF, 10 ng/mL FGF2, and 1% N2 supplement for 7 days to determine ABCG2 enrichment. Spheroids were mechanically dissociated into single-cell suspensions, rinsed in PBS, and stained with anti-ABCG2 antibody (1:500) for 30 min. Flow cytometry was performed on a Novocyte3000 instrument (ACEA Biosciences, San Diego, CA, USA), and data were processed with Novoexpress software (version 1.6.1).

### 2.12. Cell Survival Assay

Melanoma cells were seeded in 6-well plates at 5 × 10^4^ cells per well. After 24 h, they were incubated with adipocyte-derived EVs (30 μg/mL) for 24 h and then with vemurafenib (0.1 μM) for 96 h. Cells were then collected and counted using a Luna automated cell counter following staining with 0.4% Trypan Blue (1:1 *v*/*v*).

### 2.13. Determination of Cell Death Rates

Melanoma cells were seeded in 6-well plates at 5 × 10^4^ cells per well. After 24 h, they were incubated with adipocyte-derived EVs (30 μg/mL) for 24 h and then with vemurafenib (0.1 μM) for 96 h. Cell death rates were assessed by using LIVE/DEAD Viability/Cytotoxicity Kit (Thermo Fisher Scientific) and a Novocyte3000 flow cytometer. Data were processed with Novoexpress software (version 1.6.1).

### 2.14. Determination of Mitochondrial Mass and Activity

Melanoma cells were seeded in 6-well plates at 5 × 10^4^ cells per well. After 24 h, they were incubated with adipocyte-derived EVs (30 μg/mL) for 24 h. Mitochondrial mass and activity were determined by using MitoTracker Green FM and Orange CMTMRos (both 10 nM for 30 min, Thermo Fisher Scientific), respectively, and a Novocyte3000 flow cytometer, as previously reported [33,34]. Data were processed with Novoexpress software (version 1.6.1).

### 2.15. Determination of ATP Synthesis

Melanoma cells were seeded in 6-well plates at 5 × 10^4^ cells per well. After 24 h, they were incubated with adipocyte-derived EVs (30 μg/mL) for 24 h. ATP synthesis was determined by using ATP assay kit (GeneTex, Alton Pkwy Irvine, CA, USA) and an EnSpire Multimode Plate reader, as previously reported [28].

### 2.16. Determination of Mitochondrial ROS Generation

Melanoma cells were seeded in 6-well plates at 5 × 10^4^ cells per well. After 24 h, they were incubated with adipocyte-derived EVs (30 μg/mL) for 24 h. ROS generation was assessed by using MitoSOX Red (5 μM for 10 min, Thermo Fisher Scientific) and a Novocyte3000 flow cytometer, as previously reported [31]. Data were processed with Novoexpress software (version 1.6.1).

### 2.17. Pharmacological Treatment

Melanoma cells were seeded in 6-well plates at 5 × 10^4^ cells per well. After 24 h, they were incubated with SR-18292 (50 μM), XCT790 (10 μM) or N-acetylcysteine (NAC, 5 mM) for 3 h prior to exposure to adipocyte-derived EVs (30 μg/mL) for 24 h. Cells were then collected and used for transmigration and sphere formation assays, as described above.

### 2.18. Western Blot Analysis

Adipose and melanoma cells and EVs were lysed in RIPA buffer. Protein extracts (20 μg) were separated via SDS-PAGE, transferred onto nitrocellulose membranes, and probed with specific primary antibodies followed by HRP-conjugated secondary antibodies. Detection was conducted using enhanced chemiluminescence (Westar Etac Ultra 2.0, XLS075,0100, Cyanagen).

### 2.19. Statistical Analysis

Statistical analysis was performed using a statistical package (GraphPad Prism5, GraphPad Software, San Diego, CA, USA). Data are expressed as mean ± SEM from three independent experiments. Given the nature of the experimental design and the consistency of the variance, a normal distribution was assumed, and statistical significance was assessed using Student’s *t*-test or one-way ANOVA with post hoc correction. *p* < 0.05 was considered statistically significant.

## 3. Results

### 3.1. Characterization of Adipocyte-Derived EVs

**Figure 1:**
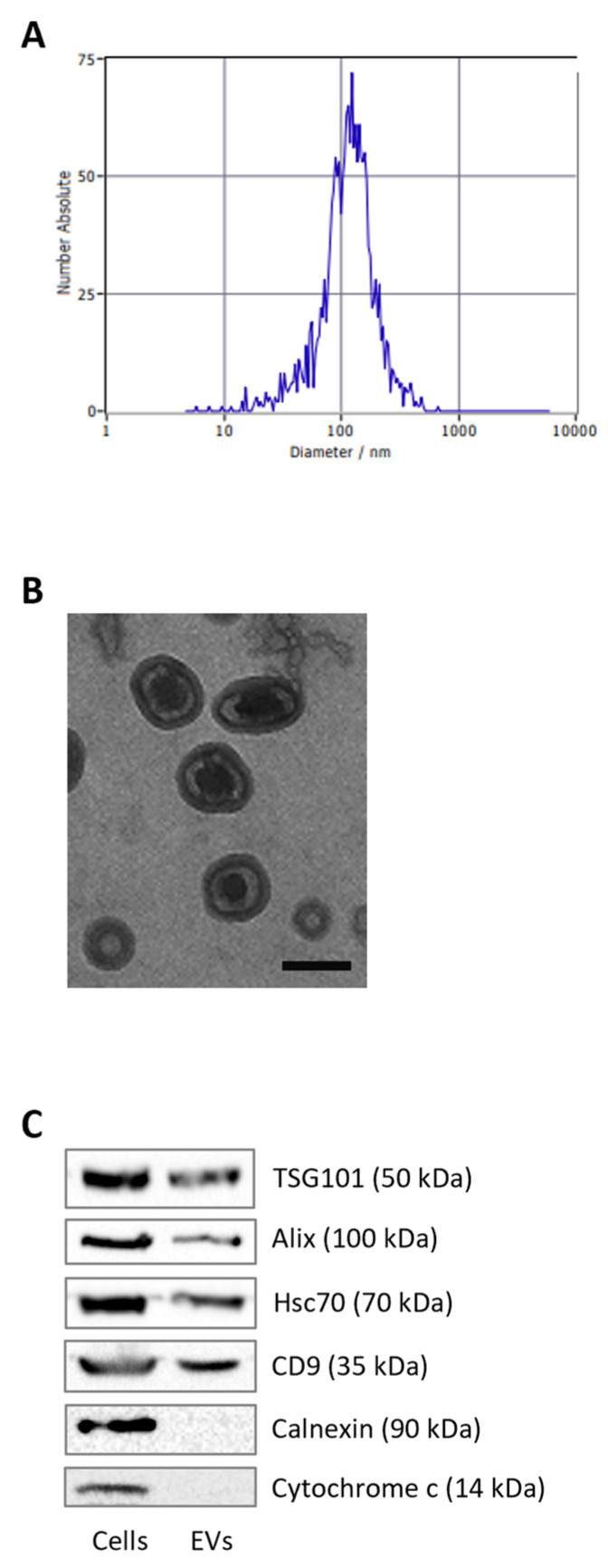
Characterization of adipocyte-derived extracellular vesicles. (**A**) Size distribution of adipocyte-derived EVs determined by nanoparticle tracking analysis. (**B**) Morphology of EVs visualized using transmission electron microscopy. Scale bar = 100 nm. (**C**) Western blot analysis showing protein levels of TSG101, Alix, Hsc70, CD9, calnexin, and cytochrome c in adipocytes and their corresponding EVs. One representative experiment out of three is shown.

Before assessing their role in melanoma progression, EVs were isolated from the conditioned media of fully differentiated 3T3-L1 cells by SEC. The vesicles obtained were then subjected to a three-pronged characterization strategy, integrating complementary approaches to validate both their physical and molecular features. NTA revealed that the majority of the isolated particles displayed an average diameter of approximately 100 nm, a size distribution consistent with that of small EVs, including exosomes (Figure 1A) [35,36,37]. TEM further confirmed these observations, providing direct visual evidence of their spherical morphology and the presence of a well-defined bilayer membrane structure, which are hallmark features of EVs (Figure 1B) [35,36,37]. In addition to morphological validation, we examined the molecular profile of the vesicles through Western blot analysis. This approach confirmed the enrichment of canonical exosomal markers, including TSG101, Alix, Hsc70, and CD9, all of which are widely recognized as indicators of small EV identity (Figure 1C) [35,36,37]. Importantly, the absence of intracellular proteins such as calnexin, a marker of the endoplasmic reticulum, and cytochrome c, a mitochondrial protein, excluded the possibility of cellular contamination, thereby supporting the specificity of our EV preparations [35,36,37]. Taken together, these findings provide strong evidence that the vesicles isolated from 3T3-L1 adipocytes represent a highly enriched population of small EVs.

### 3.2. Adipocyte-Released EVs Stimulate Melanoma Cell Migration and Invasion Without Affecting Tumor Cell Proliferation

**Figure 2:**
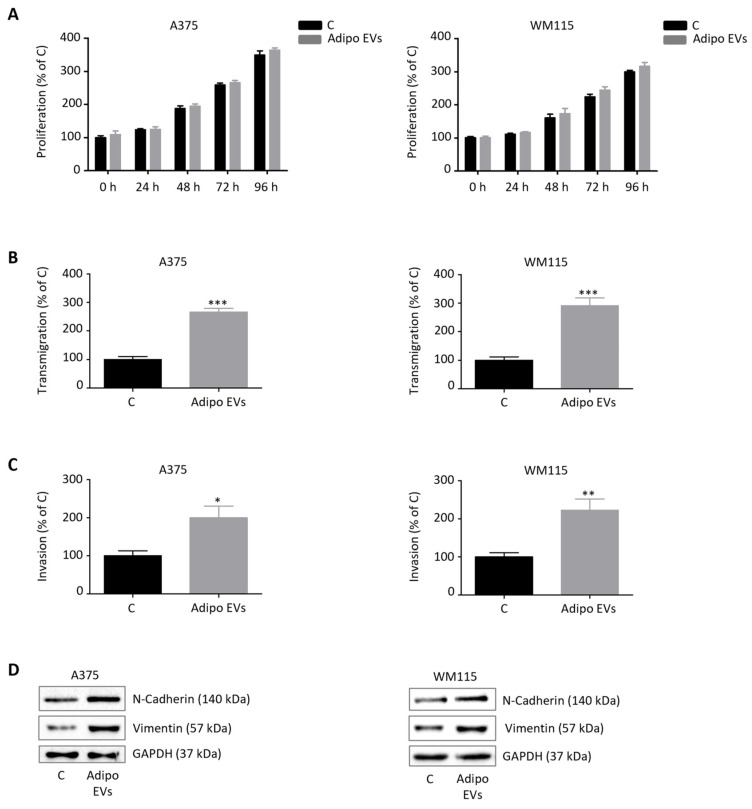
Adipocyte-released EVs stimulate melanoma cell migration and invasion without affecting tumor cell proliferation. (**A**) Adipocyte EV-treated A375 and WM115 cells (30 μg/mL, 24 h) were assessed for proliferation using a Trypan Blue exclusion assay. Data represent mean ± SEM from three independent experiments; statistical analysis was performed using a *t*-test. (**B**) Adipocyte EV-treated A375 and WM115 cells (30 μg/mL, 24 h) were assessed for migration using a transwell assay. Data represent mean ± SEM from three independent experiments; statistical analysis was performed using a *t*-test. *** *p* < 0.001 vs. A375 C or WM115 C (control). (**C**) Adipocyte EV-treated A375 and WM115 cells (30 μg/mL, 24 h) were assessed for invasion using a transwell assay. Data represent mean ± SEM from three independent experiments; statistical analysis was performed using a *t*-test. * *p* < 0.05 vs. A375 C (control), ** *p* < 0.01 vs. WM115 C (control). (**D**) Western blot analysis showing protein levels of N-cadherin and vimentin in adipocyte EV-treated A375 and WM115 cells (30 μg/mL, 24 h). GAPDH served as a loading control. One representative experiment out of three is shown.

We next investigated the functional impact of adipocyte-derived EVs on melanoma cell behavior, with particular attention to their influence on tumor growth dynamics. To this end, we employed two well-established human melanoma cell lines, A375 and WM115, both harboring the BRAF V600 mutation, which is the most common genetic alteration in this tumor [38,39,40]. This choice allowed us to assess the effects of EVs in clinically relevant, genetically comparable melanoma models while still capturing variability associated with distinct patient-derived backgrounds. Cells were exposed to EVs isolated from differentiated 3T3-L1 adipocytes. Surprisingly, treatment with these vesicles did not elicit any significant change in cell proliferation rates, as demonstrated by growth curve assays (Figure 2A). These findings suggest that adipocyte-derived EVs do not primarily function as mitogenic stimuli for melanoma cells. In contrast, a striking effect was observed on tumor cell motility and invasiveness. Both A375 and WM115 cells displayed a marked increase in migratory and invasive capacity following EV treatment (Figure 2B,C). This functional phenotype was paralleled by the activation of an epithelial-to-mesenchymal transition (EMT) program, a well-recognized process that endows tumor cells with mesenchymal traits, thereby enhancing their plasticity and metastatic potential. Molecular analyses revealed upregulation of N-cadherin, a calcium-dependent adhesion molecule typically associated with enhanced motility and cell–cell detachment, as well as vimentin, an intermediate filament protein that serves as a structural marker of mesenchymal reprogramming [41,42] (Figure 2D). The induction of these EMT markers provides mechanistic evidence that adipocyte-derived EVs can rewire the transcriptional and cytoskeletal landscape of melanoma cells to favor a more aggressive phenotype. Importantly, when melanoma cells were treated with EVs isolated from non-differentiated 3T3-L1 pre-adipocytes, no significant alterations in proliferative or migratory behavior were detected (Figure S1A,B). This observation underscores the notion that the pro-tumorigenic effects are not a generic property of stromal vesicles but are instead selectively conferred by EVs derived from mature adipocytes. Taken together, these results highlight a pivotal role for adipocyte-released EVs in promoting melanoma aggressiveness. Specifically, while these vesicles do not directly stimulate tumor cell proliferation, they act as potent modulators of melanoma cell plasticity, migration, and invasion. This selective reprogramming of malignant traits strongly supports the concept that adipocyte-derived EVs serve as critical mediators of tumor–stroma interactions within the hypodermal microenvironment, thereby facilitating metastatic dissemination.

### 3.3. Adipocyte-Associated EVs Enhance the Stem-like Traits of Melanoma Cells

**Figure 3:**
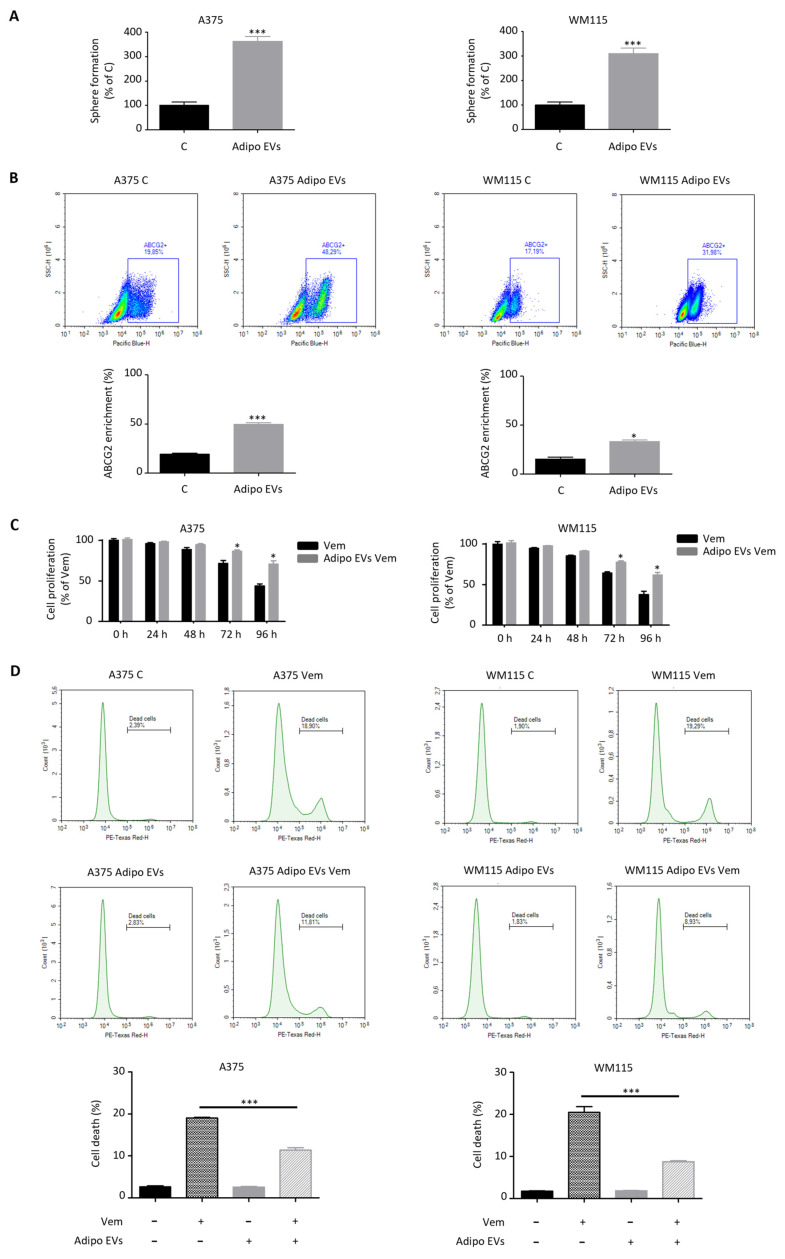
Adipocyte-associated EVs enhance the stem-like traits of melanoma cells. (**A**) Adipocyte EV-treated A375 and WM115 cells (30 μg/mL, 24 h) were assessed for spherogenic ability using a sphere formation assay. Data represent mean ± SEM from three independent experiments; statistical analysis was performed using a *t*-test. *** *p* < 0.001 vs. A375 C or WM115 C (control). (**B**) Adipocyte EV-treated A375 and WM115 cells (30 μg/mL, 24 h) were assessed for ABCG2 enrichment by flow cytometry. Data represent mean ± SEM from three independent experiments; statistical analysis was performed using a *t*-test. * *p* < 0.05 vs. WM115 C (control), *** *p* < 0.001 vs. A375 C (control). (**C**) A375 and WM115 cells pre-treated with adipocyte-associated EVs (30 μg/mL, 24 h) were subsequently exposed to vemurafenib (0.1 μM) for 96 h and assessed for proliferation using a Trypan Blue exclusion assay. Data represent mean ± SEM from three independent experiments; statistical analysis was performed using a *t*-test. * *p* < 0.05 vs. A375 Vem or WM115 Vem (control). (**D**) A375 and WM115 cells pre-treated with adipocyte-associated EVs (30 μg/mL, 24 h) were subsequently exposed to vemurafenib (0.1 μM) for 96 h and assessed for survival using LIVE/DEAD Viability/Cytotoxicity Kit. Each experiment was repeated three times. Data represent mean ± SEM from three independent experiments; statistical analysis was performed using one-way ANOVA followed by Bonferroni’s test. *** *p* < 0.001.

EMT is not only a central event in metastatic dissemination but has also been increasingly associated with the acquisition, maintenance, and expansion of cancer stem cells (CSCs) [43,44]. These stem-like subpopulations are thought to play a pivotal role in sustaining tumor heterogeneity, promoting long-term tumorigenicity, and driving resistance to conventional and targeted therapies [43,44]. Building upon this conceptual framework, we next examined whether stromal EVs derived from adipocytes could influence CSC-like traits in melanoma cells. Notably, exposure to stromal EVs significantly enhanced the spherogenic potential of both A375 and WM115 cell lines, a functional property widely recognized as a surrogate marker of stemness in cancer biology [45] (Figure 3A). At the molecular level, these phenotypic changes were closely associated with an upregulation of ATP-binding cassette subfamily G member 2 (ABCG2), an ABC transporter implicated in multidrug resistance (MDR) and previously validated as a reliable indicator of stem-like features in these melanoma cell models [34,46,47] (Figure 3B); its enrichment following EV treatment provides compelling evidence that adipocyte-derived particles contribute to the establishment of a drug-tolerant phenotype in melanoma. Functionally, this reprogramming had direct therapeutic consequences. Indeed, melanoma cells pre-treated with adipocyte-derived EVs exhibited a pronounced reduction in sensitivity to vemurafenib, a clinically relevant BRAF inhibitor, displaying markedly decreased cell death rates upon drug administration (Figure 3C,D). This finding highlights a critical role for stromal vesicles in conferring adaptive resistance to targeted therapy, underscoring their contribution to tumor resilience and progression under pharmacological pressure. Importantly, these effects were absent when melanoma cells were incubated with vesicles isolated from non-differentiated 3T3-L1 pre-adipocytes. In this case, no significant alterations in spherogenic capacity or vemurafenib response were observed (Figure S1C,D), thereby confirming that the observed pro-stemness and drug-resistance phenotypes are specifically mediated by EVs released from mature adipocytes rather than being a general property of stromal vesicles. Taken together, these data demonstrate that adipocyte-associated EVs act as powerful modulators of melanoma stem cell machinery. By simultaneously enhancing CSC traits, upregulating MDR pathways, and reducing drug sensitivity, they contribute to a more aggressive and therapy-resistant tumor phenotype. These observations not only expand our understanding of adipocyte–tumor crosstalk but also suggest that adipocyte-derived EVs may represent a novel therapeutic target to overcome CSC-driven resistance mechanisms in melanoma.

### 3.4. Adipocyte-Secreted EVs Trigger PGC-1α–Dependent Mitochondrial Biogenesis in Melanoma Cells

**Figure 4:**
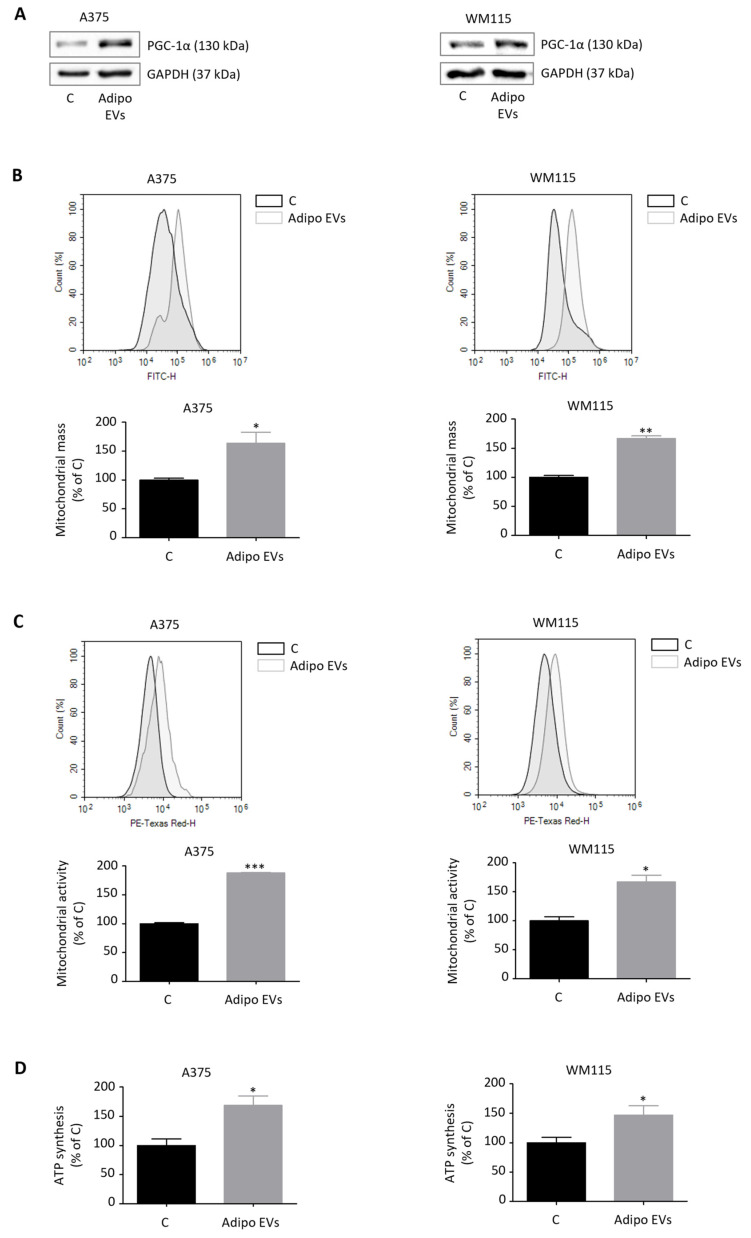
Adipocyte-secreted EVs trigger PGC-1α–dependent mitochondrial biogenesis in melanoma cells. (**A**) Western blot analysis showing protein levels of PGC-1α in adipocyte EV-treated A375 and WM115 cells (30 μg/mL, 24 h). GAPDH served as a loading control. One representative experiment out of three is shown. (**B**) Adipocyte EV-treated A375 and WM115 cells (30 μg/mL, 24 h) were assessed for mitochondrial mass by flow cytometry. Data represent mean ± SEM from three independent experiments; statistical analysis was performed using a *t*-test. * *p* < 0.05 vs. A375 C (control), ** *p* < 0.01 vs. WM115 C (control). (**C**) Adipocyte EV-treated A375 and WM115 cells (30 μg/mL, 24 h) were assessed for mitochondrial activity by flow cytometry. Data represent mean ± SEM from three independent experiments; statistical analysis was performed using a *t*-test. * *p* < 0.05 vs. WM115 C (control), *** *p* < 0.001 vs. A375 C (control). (**D**) Adipocyte EV-treated A375 and WM115 cells (30 μg/mL, 24 h) were assessed for ATP synthesis by colorimetric assay. Data represent mean ± SEM from three independent experiments; statistical analysis was performed using a *t*-test. * *p* < 0.05 vs. A375 C (control) or WM115 C (control).

**Figure 5:**
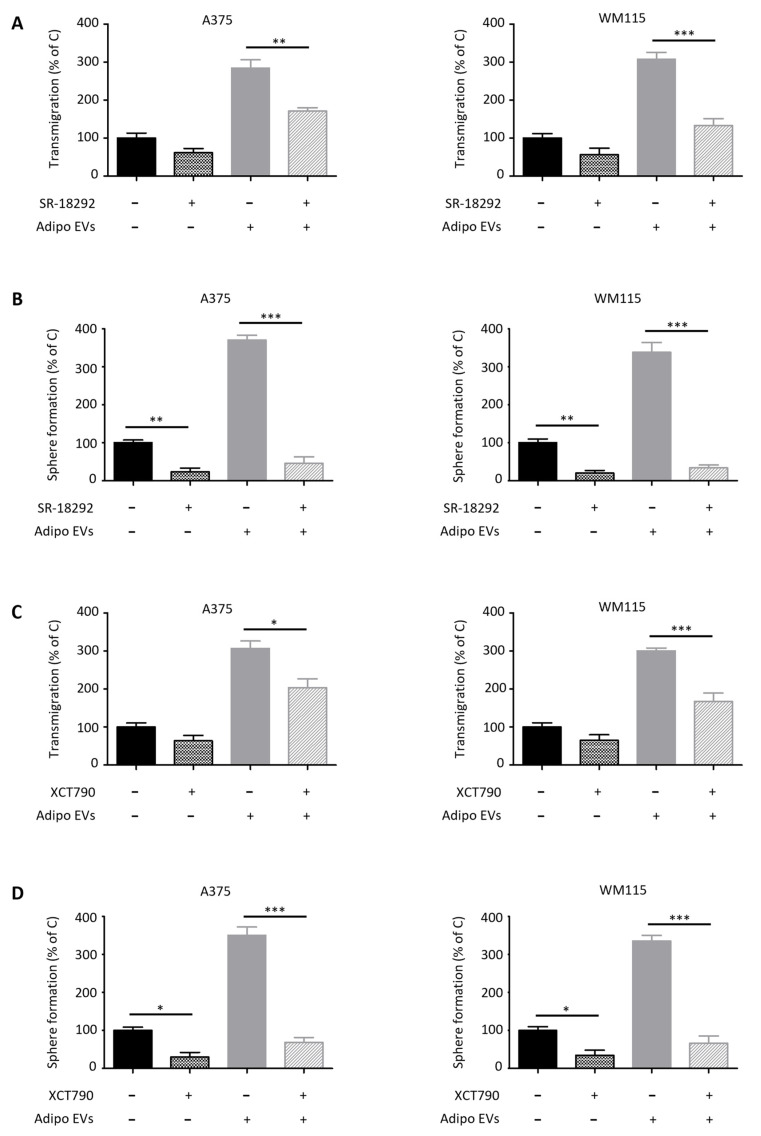
Inhibition of PGC-1α counteracts the pro-tumor activity of adipocyte-related EVs. (**A**) A375 and WM115 cells pre-treated with SR-18292 (50 μM, 3 h) were subsequently exposed to EVs (30 μg/mL, 24 h) and assessed for migration using a transwell assay. Data represent mean ± SEM from three independent experiments; statistical analysis was performed using one-way ANOVA followed by Bonferroni’s test. ** *p* < 0.01, *** *p* < 0.001. (**B**) A375 and WM115 cells pre-treated with SR-18292 (50 μM, 3 h) were subsequently exposed to EVs (30 μg/mL, 24 h) and assessed for spherogenic ability using a sphere formation assay. Data represent mean ± SEM from three independent experiments; statistical analysis was performed using one-way ANOVA followed by Bonferroni’s test. ** *p* < 0.01, *** *p* < 0.001. (**C**) A375 and WM115 cells pre-treated with XCT790 (10 μM, 3 h) were subsequently exposed to EVs (30 μg/mL, 24 h) and assessed for migration using a transwell assay. Data represent mean ± SEM from three independent experiments; statistical analysis was performed using one-way ANOVA followed by Bonferroni’s test. * *p* < 0.05, *** *p* < 0.001. (**D**) A375 and WM115 cells pre-treated with XCT790 (10 μM, 3 h) were subsequently exposed to EVs (30 μg/mL, 24 h) and assessed for spherogenic ability using a sphere formation assay. Data represent mean ± SEM from three independent experiments; statistical analysis was performed using one-way ANOVA followed by Bonferroni’s test. * *p* < 0.05, *** *p* < 0.001.

PGC-1α is a transcriptional coactivator that plays a central role in regulating mitochondrial biogenesis [48,49]. By orchestrating the transcription of nuclear-encoded mitochondrial proteins and coordinating the replication of mitochondrial DNA, PGC-1α promotes both the synthesis of new mitochondrial components and the generation of functional organelles [48,49]. We have previously shown that PGC-1α-driven mitochondrial biogenesis is not merely a metabolic adaptation but also a determinant of melanoma stem cell (SC) plasticity, where it sustains stem-like traits and contributes to tumor aggressiveness [34]. Based on these premises, we investigated whether adipocyte-secreted EVs could modulate mitochondrial dynamics in melanoma cells. Our results revealed that treatment of A375 and WM115 melanoma cell lines with adipocyte-derived vesicles induced a robust upregulation of mitochondrial biogenesis, as evidenced by a PGC-1α–dependent increase in mitochondrial mass and activity as well as ATP synthesis (Figure 4A–D). These findings highlight a novel functional link between adipocyte-derived signals and the metabolic reprogramming of melanoma cells, reinforcing the idea that mitochondrial plasticity is an integral component of tumor–stroma interactions. To further dissect the role of PGC-1α in mediating these effects, melanoma cells were co-treated with pharmacological inhibitors targeting this pathway. Specifically, SR-18292, a small molecule that promotes PGC-1α acetylation and thereby reduces its transcriptional activity, and XCT790, an established inhibitor of estrogen-related receptor alpha (ERRα), a key binding partner of PGC-1α, were employed [34]. Strikingly, inhibition of PGC-1α completely abrogated the pro-tumorigenic influence of adipocyte-derived EVs, suppressing the increase in both migratory potential and CSC-related traits (Figure 5A–D). These results provide compelling evidence that mitochondrial function, driven by PGC-1α signaling, is a critical mediator of the phenotypic changes induced by adipocyte-derived vesicles. In line with our earlier observations, EVs obtained from non-differentiated pre-adipocytes failed to elicit similar responses. No significant changes in mitochondrial biogenesis or activity were observed in melanoma cells treated with pre-adipocyte vesicles (Figure S2A–D). This reinforces the specificity of the adipocyte–melanoma interaction and underscores the notion that mature adipocytes release unique vesicular cargoes capable of reprogramming melanoma metabolism and enhancing malignancy. Altogether, these findings position PGC-1α as a central hub through which adipocyte-derived EVs exert their tumor-promoting effects. By coupling stemness induction with mitochondrial reprogramming, these vesicles enable melanoma cells to acquire greater adaptability, invasiveness, and resistance to therapy. From a translational perspective, the fact that pharmacological inhibition of PGC-1α signaling successfully blunted these effects suggests that targeting EV-induced mitochondrial biogenesis may represent a promising therapeutic avenue to counteract melanoma progression.

### 3.5. EVs from Adipocytes Induce ROS Overproduction in Melanoma Cells

**Figure 6:**
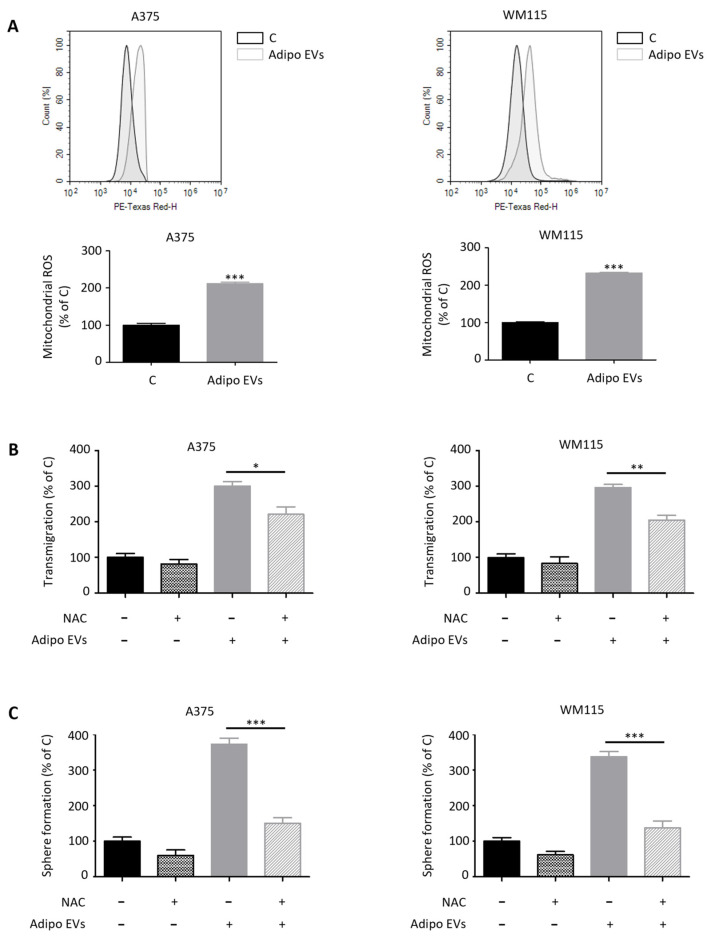
EVs from adipocytes induce ROS overproduction in melanoma cells. (**A**) Adipocyte EV-treated A375 and WM115 cells (30 μg/mL, 24 h) were assessed for mitochondrial ROS by flow cytometry. Data represent mean ± SEM from three independent experiments; statistical analysis was performed using a *t*-test. *** *p* < 0.001 vs. A375 C or WM115 C (control). (**B**) A375 and WM115 cells pre-treated with NAC (5 mM, 3 h) were subsequently exposed to EVs (30 μg/mL, 24 h) and assessed for migration using a transwell assay. Data represent mean ± SEM from three independent experiments; statistical analysis was performed using one-way ANOVA followed by Bonferroni’s test. * *p* < 0.05, ** *p* < 0.01. (**C**) A375 and WM115 cells pre-treated with NAC (5 mM, 3 h) were subsequently exposed to EVs (30 μg/mL, 24 h) and assessed for spherogenic ability using a sphere formation assay. Data represent mean ± SEM from three independent experiments; statistical analysis was performed using one-way ANOVA followed by Bonferroni’s test. *** *p* < 0.001.

Mitochondria are the primary intracellular sites of ROS production, largely through electron leakage from the respiratory chain. As such, both the abundance and functional state of these organelles exert a profound influence on the maintenance of cellular redox homeostasis [50,51]. Given our observation that adipocyte-derived EVs promote mitochondrial biogenesis and activity in melanoma cells, we next sought to determine whether these vesicles also impact ROS generation, thereby linking metabolic rewiring to redox regulation. Indeed, stromal EV-treated A375 and WM115 melanoma cells exhibited a significant increase in mitochondrial ROS production, as revealed by flow cytometry-based assays (Figure 6A). This rise in oxidative stress is consistent with the elevated mitochondrial mass and heightened respiratory activity observed upon EV exposure, further underscoring the coupling between metabolic remodeling and redox imbalance. Importantly, this redox shift is not a passive by-product but appears to be functionally relevant to tumor biology, sustaining cancer development and progression across a variety of malignancies [52,53]. To test the contribution of ROS to EV-mediated tumor aggressiveness, melanoma cells were co-treated with NAC, a potent antioxidant that replenishes intracellular glutathione pools and scavenges free radicals [54]. Notably, NAC administration markedly reduced the pro-tumorigenic effects of adipocyte-derived vesicles, effectively reversing the enhanced migratory and spherogenic potential of A375 and WM115 cells (Figure 6B,C). These results demonstrate a causal relationship between EV-induced mitochondrial ROS production and the acquisition of malignant phenotypes, highlighting ROS as a critical effector downstream of adipocyte–tumor communication. As observed in earlier experiments, pre-adipocyte-derived EVs did not elicit such redox alterations, with melanoma cells exposed to these vesicles displaying no significant increase in mitochondrial ROS levels (Figure S2E). Taken together, these findings reveal that adipocyte-secreted EVs endow melanoma cells with malignant traits by profoundly reshaping their mitochondrial hub. By simultaneously stimulating mitochondrial biogenesis and increasing ROS production, these vesicles push melanoma cells into a metabolic state that supports invasion, stemness, and resistance to therapy. Remarkably, the reversal of these effects by antioxidant treatment underscores the therapeutic potential of targeting redox vulnerabilities in EV-driven tumor progression.

## 4. Discussion

Melanoma remains the deadliest cutaneous malignancy, primarily due to its high metastatic potential and the rapid, often asymptomatic development of systemic dissemination [1,2]. Despite advances in targeted therapies and immunotherapy, disease relapse and resistance remain frequent, underscoring the urgent need for a deeper understanding of the mechanisms that regulate melanoma progression [5,6]. In this context, the tumor microenvironment has emerged as a critical determinant of melanoma aggressiveness [55,56]. Among its various stromal components, adipose tissue plays a pivotal role, particularly in the dermis and hypodermis, where tumor cells first encounter adipocytes during vertical invasion [8,27]. While previous studies have highlighted a complex crosstalk between melanoma and adipose cells, this dialog has largely been attributed to soluble factors such as cytokines and metabolites [5,6,7,8,9]. However, growing evidence suggests that EVs represent an equally, if not more, important mediator of intercellular communication, with the potential to reprogram tumor behavior and promote disease progression [20,21].

In the present study, we sought to clarify the functional relevance of adipocyte-derived EVs in melanoma biology by evaluating their impact on two human melanoma cell lines, A375 and WM115. Our findings reveal that adipocyte-secreted vesicles stimulate melanoma cell migration and invasion without affecting proliferation, indicating that these vesicles primarily contribute to tumor dissemination rather than growth. Mechanistically, this effect was associated with the activation of an EMT program, as evidenced by increased expression of N-cadherin and vimentin, two canonical EMT markers. These results not only underscore the role of stromal EVs in accelerating melanoma progression but also align with similar observations reported in breast, prostate, and lung cancers, where tumor–stroma vesicular interactions have been shown to enhance metastatic behavior [28,57,58,59].

Beyond their effect on migration and invasion, adipocyte-derived EVs were found to promote the emergence of a stem-like phenotype in melanoma cells, conferring resistance to the BRAF inhibitor vemurafenib. This phenotype was characterized by enhanced sphere-forming ability and upregulation of ABCG2, a multidrug resistance transporter and well-established CSC marker [34,46,47]. The acquisition of stem-like traits and drug resistance highlights a novel mechanism through which adipocyte–melanoma communication fosters tumor resilience. While La Camera and colleagues previously described a role for adipose-derived vesicles in supporting CSC populations in breast cancer [58], our study provides, to the best of our knowledge, the first direct evidence that stromal EVs can elicit CSC properties in melanoma. This expands the paradigm of adipocyte–tumor crosstalk to include vesicular regulation of melanoma plasticity and therapeutic resistance.

A growing body of literature has identified increased mitochondrial mass and activity as distinctive features of CSCs across several malignancies, including melanoma [60,61,62]. In this tumor type, mitochondrial biogenesis relies heavily on the transcriptional coactivator PGC-1α, which orchestrates the expression of genes involved in oxidative metabolism [34,62]. Consistent with this, we demonstrated that adipocyte-derived EVs could trigger PGC-1α-dependent mitochondrial biogenesis in melanoma cells, resulting in elevated mitochondrial mass and activity as well as ATP production. Importantly, pharmacological inhibition of PGC-1α with SR-18292, or blockade of its functional partner ERRα with XCT790, abrogated the pro-tumor effects of adipocyte-derived EVs, suppressing both migration and stem-like traits. These results strongly suggest that mitochondrial reprogramming is not a mere by-product of EV signaling but a central driver of the phenotypic changes observed. Moreover, they are in agreement with recent findings showing that adipocyte-derived vesicles promote melanoma metabolism via fatty acid oxidation (FAO) [63]. Collectively, our data point to the PGC-1α axis as a critical mediator of adipocyte–melanoma interactions and highlight mitochondrial metabolism as a potential therapeutic target.

ROS production represents another crucial downstream consequence of mitochondrial reprogramming [50,51]. In cancer cells, mitochondrial ROS act as signaling molecules that amplify tumorigenic pathways, promote genomic instability, and accelerate metastatic progression [52,53]. We and others have previously identified ROS overproduction as a hallmark of melanoma stem-like cells [34,64]. Here, we observed that stromal EVs induce a significant increase in mitochondrial ROS levels in melanoma cells, linking vesicle-driven mitochondrial rewiring to redox imbalance. Notably, treatment with the antioxidant NAC markedly reduced the pro-tumorigenic effects of EVs, highlighting the functional importance of ROS in mediating the observed phenotypic alterations. This finding reinforces the notion that redox homeostasis represents a vulnerable node in EV-driven melanoma progression and suggests that antioxidants or redox-targeting drugs may have therapeutic value in disrupting adipocyte–tumor interactions.

While our findings provide valuable insights, the study has certain limitations. All experiments were conducted in vitro, which, although informative for understanding cellular mechanisms, cannot fully replicate the complexity of the tumor microenvironment in vivo. Future studies employing relevant animal models will be crucial to validate the physiological and clinical significance of adipocyte-derived EVs in melanoma progression [65]. Additionally, although we highlighted PGC-1α and ROS as important mediators of EV-induced effects, the exact molecular cargo responsible for these changes remains unclear. Potential contributors include microRNAs, lipid molecules, and specific proteins, all of which have previously been linked to EV-driven tumorigenesis [17,18,19]. Importantly, EVs may also carry PGC-1α itself or mitochondrial components, which could be directly transferred to melanoma cells to enhance mitochondrial biogenesis, oxidative metabolism, and stem-like phenotypes; such direct transfer could represent a novel mechanism by which stromal cells modulate tumor aggressiveness and metabolic plasticity [66,67]. Identifying and characterizing these molecular drivers will not only expand our understanding of adipose–tumor interactions but could also uncover novel biomarkers and therapeutic targets for combating melanoma.

## 5. Conclusions

In conclusion, our study provides novel mechanistic insights into how adipocyte-derived EVs contribute to melanoma progression. By promoting EMT, inducing a CSC-like phenotype, activating PGC-1α-driven mitochondrial biogenesis, and enhancing ROS production, these vesicles reprogram melanoma cells toward a more invasive, stem-like, and drug-resistant state. Importantly, pharmacological and antioxidant interventions were able to suppress these effects, indicating that targeting EV-induced mitochondrial and redox pathways may represent a promising therapeutic strategy. Overall, these findings expand the understanding of melanoma–stroma interactions and position adipocyte-derived EVs as key modulators of tumor aggressiveness and potential targets for future therapeutic interventions.

## Data Availability

The original contributions presented in this study are included in the article/Supplementary Materials. Further inquiries can be directed to the corresponding author(s).

## Supplementary Materials

The following supporting information can be downloaded at: https://www.mdpi.com/article/10.3390/antiox15030333/s1, Figure S1: Extracellular vesicles from pre-adipocytes do not affect melanoma cell phenotype; Figure S2: Extracellular vesicles from pre-adipocytes do not alter melanoma cell mitochondrial metabolism.
