# Supplementary material for: Adipocyte-Derived Extracellular Vesicles Endow Melanoma Cells with Stem-like Traits via PGC-1α–Mediated Mitochondrial Reprogramming
Source: Antioxidants (Basel). 2026 Mar 6;15(3):333. doi: 10.3390/antiox15030333 (PMC13024706; doi:10.3390/antiox15030333)
Supplement: Supplementary file 1 [file antioxidants-15-00333-s001.zip › antioxidants-4120699-supplementary.pdf]

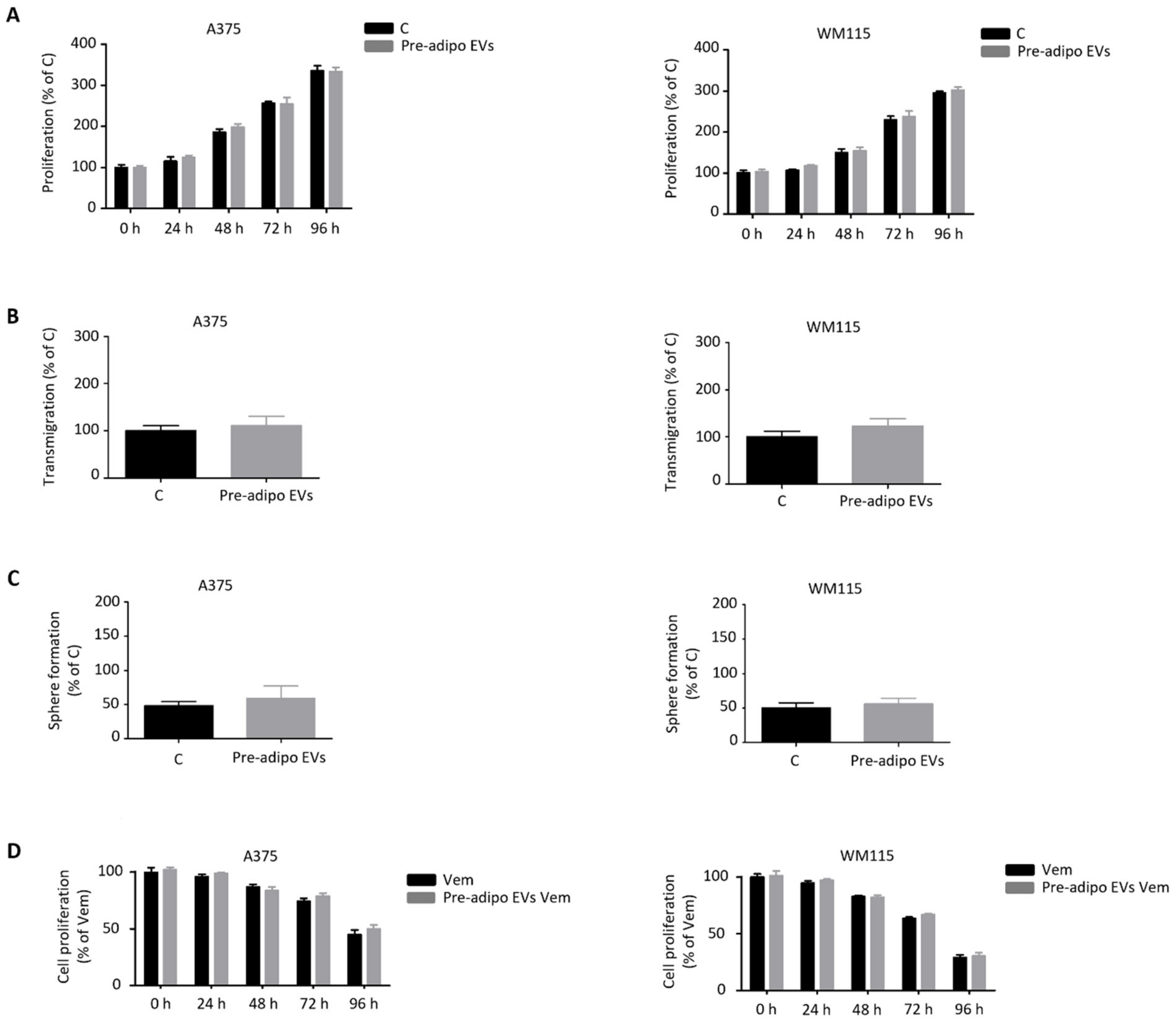

Figure S1. Extracellular vesicles from pre-adipocytes do not affect melanoma cell phenotype. A, Pre-adipocyte EV-treated A375 and WM115 cells (30 µg/mL, 24 hours) were assessed for proliferation using Trypan Blue exclusion assay. Data represent mean ± SEM from three independent experiments; statistical analysis was performed using a t-test. **B**, pre-adipocyte EV-treated A375 and WM115 cells (30 µg/mL, 24 hours) were assessed for migration using transwell assay. Data represent mean ± SEM from three independent experiments; statistical analysis was performed using a t-test. **C**, Pre-adipocyte EV-treated A375 and WM115 cells (30 µg/mL, 24 hours) were assessed for spherogenic ability using sphere formation assay. Data represent mean ± SEM from three independent experiments; statistical analysis was performed using a t-test. **D**, A375 and WM115 cells pre-treated with pre-adipocyte-associated EVs (30 µg/mL, 24 hours) were subsequently exposed vemurafenib (0.1 µM) for 96 hours and assessed for proliferation using Trypan Blue exclusion assay. Data represent mean ± SEM from three independent experiments; statistical analysis was performed using a t-test.

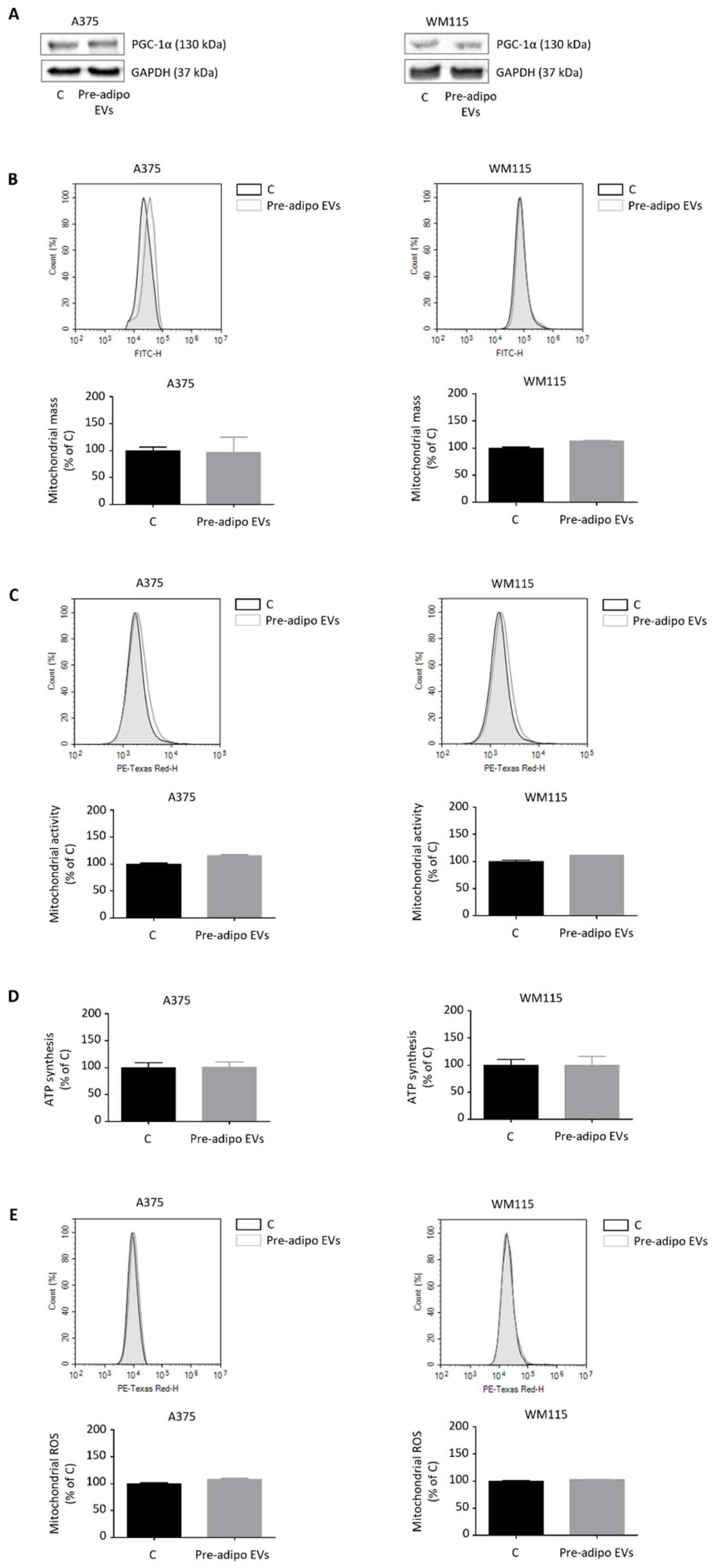

Figure S2. Extracellular vesicles from pre-adipocytes do not alter melanoma cell mitochondrial metabolism. A, Western blot analysis showing protein levels of PGC1-α in pre-adipocyte EV-treated A375 and WM115 cells (30 µg/mL, 24 hours). GAPDH served as a loading control. One representative experiment out of three is shown. **B**, Pre-adipocyte

EV-treated A375 and WM115 cells (30 µg/mL, 24 hours) were assessed for mitochondrial mass by flow cytometry. Data represent mean ± SEM from three independent experiments; statistical analysis was performed using a t-test. **C**, Pre-adipocyte EV-treated A375 and WM115 cells (30 µg/mL, 24 hours) were assessed for mitochondrial activity by flow cytometry. Data represent mean ± SEM from three independent experiments; statistical analysis was performed using a t-test. **D**, Pre-adipocyte EV-treated A375 and WM115 cells (30 µg/mL, 24 hours) were assessed for ATP synthesis by colorimetric assay. Data represent mean ± SEM from three independent experiments; statistical analysis was performed using a t-test. **E**, Pre-adipocyte EV-treated A375 and WM115 cells (30 µg/mL, 24 hours) were assessed for mitochondrial ROS by flow cytometry. Data represent mean ± SEM from three independent experiments; statistical analysis was performed using a t-test.
